# Comparative study of biomarkers for the early identification of Epstein–Barr virus-associated hemophagocytic lymphohistiocytosis in infectious mononucleosis

**DOI:** 10.1186/s12879-023-08654-6

**Published:** 2023-10-26

**Authors:** Lisha Cai, Yuan Xing, Yahong Xia, Zihan Zhang, Zebin Luo, Yongmin Tang, Yan Chen, Xiaojun Xu

**Affiliations:** 1 Division of Hematology-Oncology, Children’s Hospital of Zhejiang University School of Medicine, No. 57 Zhugan Lane, Yan-an Street, Hangzhou, 310003 PR China; 2 Department of Pediatrics, The Affiliated Hospital of Zunyi Medical University, Guizhou Children’s Hospital, No. 149 Dalian Rd, Huichuan District, Zunyi, Guizhou 563000 PR China

**Keywords:** Hemophagocytic lymphohistiocytosis, Infectious mononucleosis, Diagnosis, Interleukin-10, Interferon-γ

## Abstract

**Background and aim:**

Epstein-Barr virus-associated hemophagocytic lymphohistiocytosis (EBV-HLH) and infectious mononucleosis (EBV-IM) share mimic symptoms in the early stages of childhood development. We aimed to examine the clinical features and laboratory indices of these two diseases in children and uncover unique indicators to assist pediatricians in identifying these diseases early.

**Methods:**

We collected clinical data from 791 pediatric patients diagnosed with EBV-IM or EBV-HLH, compared the clinical traits and laboratory biomarkers presented in the two groups, and constructed predictive models based on them.

**Results:**

Patients with EBV-IM had greater ratios of cervical lymphadenopathy, eyelid edema, and tonsillitis, whereas individuals with EBV-HLH were more likely to have hepatomegaly and splenomegaly. When using the criteria of interleukin (IL)-10 > 89.6 pg/mL, interferon (IFN)-γ > 45.6 pg/mL, ferritin > 429 μg/L, D-dimer > 3.15 mg/L and triglycerides > 2.1 mmol/L, the sensitivity was 87.9%, 90.7%, 98.1%, 91.1% and 81.5% to predict EBV-HLH, while the specificity was 98.4%, 96.3%, 96.5%, 94.1% and 80.6%, respectively. A logistic regression model based on four parameters (IL-10, ferritin, D-dimer, and triglycerides) was established to distinguish EBV-HLH patients from EBV-IM patients, with a sensitivity of 98.0% and a specificity of 98.2%.

**Conclusions:**

IL-10, IFN-γ, ferritin and D-dimer levels are significantly different between EBV-HLH and EBV-IM. Predictive models based on clinical signs and laboratory findings provide simple tools to distinguish the two situations.

**Supplementary Information:**

The online version contains supplementary material available at 10.1186/s12879-023-08654-6.

## Introduction

Epstein-Barr virus (EBV) is a subfamily herpesvirus that over 90% of individuals have contracted and is one of eight major herpesviruses that cause human disease [1, 2]. EBV infection in humans can be asymptomatic. However, most patients develop infectious mononucleosis (IM) after infection, while other types like EBV-associated hemophagocytic lymphohistiocytosis (EBV-HLH), chronic active EBV infection (CAEBV), EBV-associated lymphoproliferative disorder (EBV-LPD) are relatively rare. In more extreme circumstances, EBV infection can also lead to the development of malignancies such as lymphoma, nasopharyngeal carcinoma, and gastric cancer [3, 4].

Fever, tonsillitis, and cervical lymph node enlargement are clinical symptoms of IM, which can affect people of all ages and appear in all populations [5]. The clinical signs and symptoms of IM vary and lack specificity. Patients do not always produce EBV-specific antibodies when infected with the virus for the first time and do not show typical clinical symptoms if they have immunosuppression or genetic abnormalities, potentially resulting in an incorrect diagnosis [6]. A set of hyperinflammatory response syndromes known as hemophagocytic lymphohistiocytosis(HLH) are characterized by overactive cytotoxic T cells (CTL) and macrophages [7]. EBV-HLH is defined as HLH with EBV infection and no gene abnormalities associated with primary HLH [8]. EBV-HLH has a higher fatality rate than EBV-IM; however, the two illnesses occasionally exhibit similar early-stage symptoms. Thus, early identifying and treating these two diseases is critical for saving lives [9]. Our team has used a cytokine profile as a supplementary instrument for rapidly evaluating HLH [10, 11], and it is essential to discover other clinical or laboratory markers that aid in the early identification of HLH in patients with childhood EBV-IM in addition to the cytokine pattern. Li et al. and Smits et al. have developed predict models to early identify HLH patients from those with acute EBV-infection or suspected HLH, which include clinical, biologic, and cytologic variables but without cytokine profile [12, 13].

Herein, a retrospective study of pediatric patients diagnosed with EBV-HLH and EBV-IM was conducted to analyze clinical characteristics and laboratory data to identify potential indices to predict EBV-HLH. In addition, we investigated whether the simultaneous detection of multiple biological indicators could improve the accuracy of the differential diagnosis.

## Patients and Methods

### Patients

Pediatric patients with EBV-IM at Children’s Hospital of Zhejiang University School of Medicine between January 2019 and March 2022 were included in this retrospective analysis, as were those diagnosed with EBV-HLH between June 2017 and December 2022. Clinical data included sex, age, physical signs, routine blood analysis results, serum cytokines (interleukin (IL)- 6, IL-10, interferon-gamma (IFN-γ) and tumor necrotic factor-alpha (TNF-α)), lymphocyte subsets, and EBV DNA copies. The study protocol was approved by the Ethics Committee of Children’s Hospital of Zhejiang University School of Medicine (IRB number 2023-IRB-0014-P-01) and conducted in accordance with the tenets of the Declaration of Helsinki.

### Diagnostic criteria

EBV-HLH was diagnosed in patients who match the HLH-2004 criteria and show signs of active EBV infection [14]. Patients fulfilled at least five of the eight criteria were diagnosed as HLH: (1) fever; (2) splenomegaly; (3) at least two of the three lineages are affected by cytopenia (hemoglobin < 90 g/L, platelets < 100 × 10^9^/L, and/or neutrophils < 1.0 × 10^9^/L); (4) hypertriglyceridemia (≥ 265 mg/dL) and/or hypofibrinogenemia (≤ 150 g/dL); (5) hematophagy has been discovered in the bone marrow, spleen, liver, and lymph nodes; (6) natural killer (NK) cell activity is low or undetectable; (7) ferritin levels ≥ 500 μg/L; and (8) increased interleukin-2 receptor levels (soluble CD25). Patients who were positive for seral EBV DNA by polymerase chain reaction (PCR) were considered as active EBV infection. Patients fulfilling the definition of primary HLH were excluded. Primary HLH was defined as presence of genetic inborn errors of immunity with HLH as a main feature of the disease, including FHL and HLH caused by RAB27A, LYST, AP3B1, SH2D1A, and BIRC4 variants.

EBV-IM is diagnosed on the basis of clinical manifestation and laboratory results [15]. The clinical indices include the following: (1) fever; (2) pharyngeal tonsillitis; (3) cervical lymph node enlargement; (4) splenomegaly; (5) hepatomegaly; and (6) eyelid edema. The biological marker findings in EBV-IM are as follows: (1) Positive for anti-EBV-VCA-IgM and anti-EBV-VCA-IgG antibodies and negative for anti-EBV-NA-IgG antibodies; (2) negative for anti-EBV-VCA-IgM antibodies but positive for anti-EBV-VCA-IgG and low-affinity antibodies; (3) anti-EBV-VCA-IgG levels increased ≥  4-fold in two serum samples; and (4) positive for EBV DNA by polymerase chain reaction. Patients who met one of the biochemical indicators and any three physical indices were diagnosed with IM.

### Determination of cytokines and lymphocyte subsets

Cytokine levels were determined by flow cytometry using a cytometric bead array (CBA) human Th1/Th2 Cytokine Kit II (BD, USA) [16]. The lower and upper limits of detection for each cytokine were 1.0 and 5000 pg/mL, respectively. In the statistical analysis, 5000 pg/mL was substituted when values exceeded this number. Peripheral blood samples were examined using a Multitest TBNK kit (BD, USA) and FACSDiva software (BD, USA) on a FACSCanto II (Becton–Dickinson, USA) for the lymphocyte subset assay.

### Statistical method

Proper quartiles (median, Q1, and Q3), or absolute values and percentages were used to illustrate the data. The two datasets were compared using the chi-squared test or the Mann–Whitney U test. The effectiveness of biomarkers in distinguishing EBV-HLH from EBV-IM was assessed using receiver operating characteristic (ROC) curves. The optimal cutoff values were selected using the Youden index. Logistic regression was used to build a model with four parameters to assess the diagnostic value in discriminating EBV-HLH and EBV-IM. The Hosmer–Lemeshow goodness of fit test was utilized to estimate how well the prediction model was calibrated. *P* > 0.05 indicated that the predictive model’s calibration level was meaningful. The data were analyzed using SPSS (20.0) and GraphPad Prism (9.4.1). *P* < 0.05 was considered to indicate significance.

## Results

### Patients’ characteristics

Of the 791 children, 108 were diagnosed with EBV-HLH and 683 with EBV-IM. The demographic characteristics for both samples are displayed in Table 1. The ratio of female patients in HLH group was higher than that in IM group (*P* = 0.011). Regarding symptoms, patients with EBV-HLH were more likely to present with hepatomegaly and splenomegaly, while those with EBV-IM more frequently presented with eyelid edema, cervical lymphadenopathy, and tonsillitis (all *P* < 0.0001).

**Table 1:** The demographic and clinical features of the 791 children with EBV-associated infectious mononucleosis (IM) and hemophagocytic lymphohistiocytosis (HLH)

	EBV-HLH	IM	χ^2^	*P*
Median age (year)	3.5 (0.4–14.0)	4.1 (1.0–14.0)	/	0.055
Male-to-Female Ratio	43/65	362/321	6.490	0.011
Fever	108/108	676/683	0.254	0.614
Hepatomegaly	97/108	440/683	27.583	< 0.0001
Splenomegaly	79/108	369/672	12.659	< 0.0001
Cervical lymphadenopathy	82/102	670/673	106.386	< 0.0001
Eyelid edema	21/108	476/683	100.822	< 0.0001
Tonsillitis	69/102	664/669	188.677	< 0.0001

### Comparison of laboratory findings in EBV-HLH and EBV-IM patients

EBV-HLH and EBV-IM can cause multiple organ damage; thus, the hematological indices, liver and renal function parameters, serum cytokines, and lymphocyte subsets were examined. Unlike in EBV-IM patients, cytopenia, hypoalbuminemia and elevated bilirubin, liver enzymes, triglycerides, ferritin, and lactate dehydrogenase (LDH) were common in patients with EBV-HLH (Table 2). D-dimer level was significantly higher in EBV-HLH than that in EBV-IM while fibrinogen levels were much lower. The median EBV-DNA copy number in the plasma was approximately 90 times higher in EBV-HLH patients than that in EBV-IM patients.

**Table 2:** Distribution of investigated laboratory parameters among pediatric patients with EBV-HLH and EBV-IM

Parameters	EBV-HLH (108)			IM (683)			
	Median	Q1, Q3	Min, Max	Median	Q1, Q3	Min, Max	*P*
White blood cell count, × 10^9^/L	1.9	1.1, 2.7	0.2, 22.9	13.8	10.4, 18.2	1.3, 169.9	< 0.0001
Platelet count, × 10^9^/L	56.0	36.3, 76.8	2.0, 217.0	205.5	162.8, 255.3	48.0, 740.0	< 0.0001
Hemoglobin g/L	91	84.3, 99.0	61.0, 127.0	118	112.0, 125.0	86.0, 150.0	< 0.0001
Neutrophil count, × 10^9^/L	0.6	0.3, 0.9	0.03, 6.1	6.9	5.0, 9.1	0.4, 22.1	< 0.0001
Lymphocyte count, × 10^9^/L	0.9	0.4, 2.0	0.02, 17.5	2.4	1.6, 3.3	0.4, 17.2	0.001
Albumin, g/L	31.0	28.1, 35.4	21.7, 53.7	37.6	35.7, 40.0	26.0, 69.3	< 0.0001
Total bilirubin, μ mol/L	19.4	7.7, 50.0	2, 135	5.8	4.1, 7.4	1.0, 60.0	< 0.0001
AST, IU/L	288.0	119.0, 496.0	31, 8872	72.0	48.0, 129.0	16, 807	< 0.0001
ALT, IU/L	141.5	67.0, 301.0	11, 3074	63.0	31.0, 157.0	3, 878	< 0.0001
LDH, IU/L	1597.5	1036.3, 2604.5	340, 12501	566.0	461.8, 748.0	34, 2419	< 0.0001
Creatinine, μ mol/L	40.0	28.0, 48.8	11, 393	47.0	34.0, 54.0	3, 122	0.996
Fib (g/L)	1.12	0.81, 1.55	0.25, 3.43	2.33	1.99,2.78	0.53,4.85	< 0.0001
IL-6 (pg/ml)	54.1	24.1, 107.4	4.8, 5000	22.2	10.9,69.7	1.7,4766.6	0.437
IL-10 (pg/ml)	499.6	203.0, 1339.8	7,5000	19.9	12.3,33.0	1.0,1359	< 0.0001
IFN-γ (pg/ml)	629.0	139.5, 1948.8	1.3, 5000	8.2	4.4,14.7	1.0,1824.6	< 0.0001
CD19 + /CD20 +	8.9	4.7, 14.3	0.7, 45.4	4.3	2.5,7.1	0.1,26.4	< 0.0001
CD3 + (%)	84.2	76.5, 88.6	51.3, 98.0	85.3	79.9,89.3	42.6,98.7	0.07
CD4 + (%)	26.5	18.2, 39.3	2.5, 69.2	17.3	12.7,23.4	1.0,60.5	< 0.0001
CD8 + (%)	44.7	35.3, 57.0	9.1, 91.3	56.0	45.6,67.0	8.7,89.7	< 0.0001
CD3- CD56 + (%)	4.3	2.4,7.2	0.4, 26.7	5.3	3.6,7.5	0.8,47.4	0.089
CD4 + /CD8 +	0.61	0.32,1.07	0.03, 2.3	0.31	0.20,0.50	0.05,2.0	< 0.0001
Ferritin μg/L	1500	1500,1500	296, 1500	113	74.4,175.6	11,1500	< 0.0001
Triglyceride (mmol/L)	3.1	2.2,4.1	0.6,20.3	1.4	1.1,2.0	0.3,15.0	< 0.0001
EBV DNA (copies/ml)	123,000	24,750, 847,500	500,3.48 × 10^7^	1340	500,6120	500,2.53 × 10^6^	0.002
D-dimer (mg/L)	12.56	5.26, 25.00	0.87, 87.83	1.00	0.64,1.63	0.10,8.00	< 0.0001

*ALT* alanine aminotransferase, *AST* aspartate aminotransferase, *LDH* lactate dehydrogenase

**Fig. 1:**
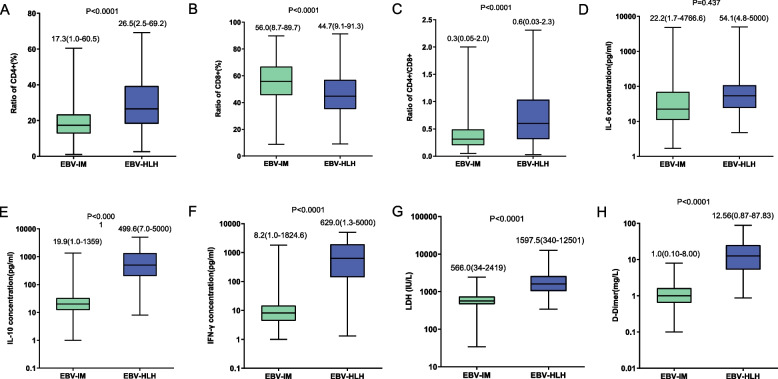
Laboratory indicators were compared between patients with EBV-HLH and those with EBV-IM, including proportion of CD4 + T cells among all T cells (**A**), proportion of CD8 + T cells (**B**), ratio of CD4 + /CD8 + T cells (**C**), levels of interleukin (IL)-6 (D), IL-10 (**E**), interferon-gamma (IFN-γ) (**F**), lactate dehydrogenase (LDH) (**G**) and D-dimer (**H**)

Regarding cytokines, the IL-6 level was similar, while IL-10 (median concentration: 499.6 pg/mL vs. 19.9 pg/mL, *P* < 0.001) and IFN-γ (median concentration: 629.0 pg/mL vs. 8.2 pg/mL, *P* < 0.001) levels were significantly higher in patients with EBV-HLH (Fig. 1). The CD8 + T cell ratio in the EBV-HLH group was considerably lower than that in the EBV-IM group (44.7% vs. 56.0%, *P* < 0.001), while the total CD3 + T cell count was comparable (84.2% vs. 85.30%, *P* = 0.07).

As the clinical manifestation and laboratory data can be affected by the age of patients, we then compared the above parameters in different age groups. The patients were divided into three groups: age ≤ 3 years old, > 3 years old and ≤ 6 years old, and > 6 years old. The ratios of CD4 + and CD8 + T cells were different among the three groups while other parameters were comparable. The comparison results were similar among the three age groups which were shown in Supplementary Figs. 1 and 2.

### Distinguishing EBV-HLH from EBV-IM by laboratory parameters

**Fig. 2:**
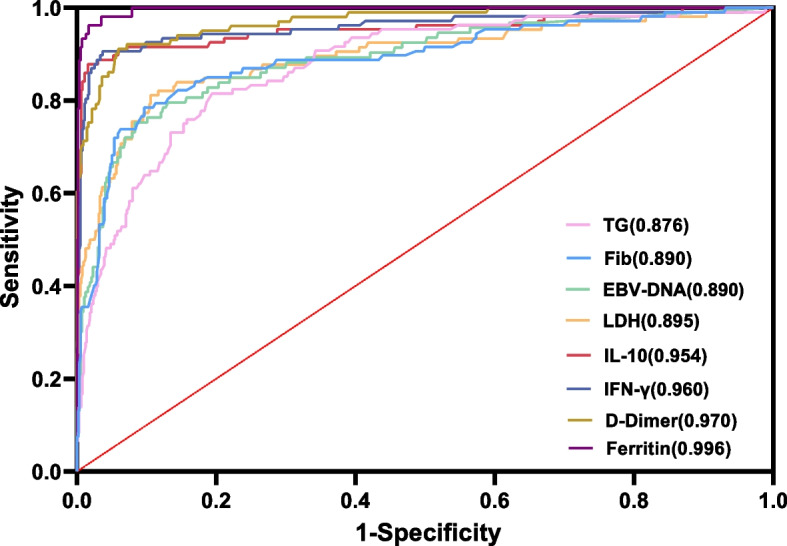
The ROC curves for EBV-HLH prediction using triglycerides (TG), fibrinogen (Fib), seral EBV-DNA loads, lactate dehydrogenase (LDH), interleukin (IL)-10 (IL-10), interferon-gamma (IFN-γ), D-dimer and ferritin

Although the severity of EBV-HLH and EBV-IM differ greatly, it is challenging to promptly recognize and distinguish EBV-HLH from EBV-IM due to a lack of early-stage symptoms. Using ROC analysis, we compared the ability of IL-10, IFN-γ, LDH, D-dimer, EBV-DNA copy number, triglycerides, fibrinogen, and ferritin to distinguish EBV-HLH from EBV-IM (Fig. 2). The areas under the curve (AUC) for IL-10, IFN-γ, LDH, D-dimer, triglycerides and ferritin were 0.954, 0.960, 0.895, 0.970, 0.876 and 0.996, respectively, indicating that these indices showed good performance for predicting EBV-HLH. When the cutoff values for IL-10, IFN-γ, LDH, D-dimer, triglycerides and ferritin were set at 89.6 pg/mL, 45.6 pg/mL, 948.5 IU/L, 3.15 mg/L, 2.1 mmol/L and 429 μg/L, the sensitivity was 87.9%, 90.7%, 81.1%, 91.1%, 81.5%and 98.1%, and the specificity was 98.4%, 96.3%, 89.4%, 94.1%, 80.6% and 96.5%, respectively (Table 3).

**Table 3:** Predictive accuracy of IL-10, IFN-γ, LDH, the D-dimer, EBV-DNA loads, Triglyceride (TG), Fibrinogen (Fib) and Ferritin for EBV-HLH and EBV-IM. The numbers in the brackets indicate the areas under curves

	Cutoff	AUC, %	Sensitivity, %	Specificity, %	PPV, %	NPV, %	Youden	P
IL-10 (pg/ml)	89.6	95.4	87.9	98.4	90.4	97.9	0.86	< 0.0001
IFN-γ (pg/ml)	45.6	96.0	90.7	96.3	80.8	98.4	0.87	< 0.0001
LDH (IU/L)	948.5	89.5	81.1	89.4	56.2	96.6	0.71	< 0.0001
D-dimer (mg/L)	3.15	97.0	91.1	94.1	73.6	98.3	0.85	< 0.0001
EBV-DNA loads (copies/ml)	26,400	89.0	75.3	91.7	59.8	95.8	0.67	< 0.0001
Triglyceride (mmol/L)	2.1	87.6	81.5	80.6	41.5	96.3	0.62	< 0.0001
Fibrinogen (g/L)	1.6	89.0	78.5	90.3	60.9	95.6	0.69	< 0.0001
Ferritin (μg/L)	429	99.6	98.1	96.5	84.6	99.6	0.95	< 0.0001

*AUC* area under the curve, *PPV* positive predictive value, *NPV* negative predictive value, *Youden* Youden’s index

**Fig. 3:**
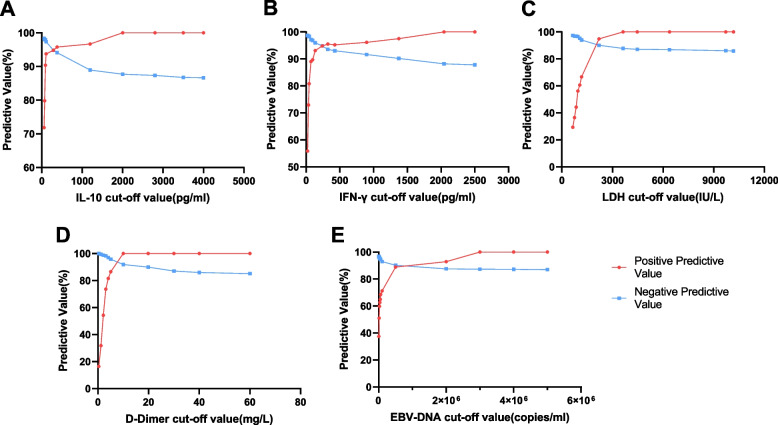
Positive and negative predictive value of partial univariate with different cutoff points. **A** interleukin (IL)-10 (IL-10); **B** interferon-gamma (IFN-γ); **C** lactate dehydrogenase (LDH); D, D-dimer; E, seral EBV-DNA load

Of the five univariate variables, IL-10, IFN-γ, LDH, D-dimer and EBV-DNA copy number, as the cutoff values increased, the positive predictive value (PPV) reached 100%, and the negative predictive value (NPV) reduced to approximately 86%. The ideal cutoff value may be the junction of PPV and NPV, which has reference value for clinically distinguishing EBV-HLH from EBV-IM (Fig. 3). The greatest cutoff value selected with the intersection points is higher than that in the maximum value Youden’s index (IL-10: 288.3 pg/mL vs. 89.6 pg/mL, IFN-γ: 240.7 pg/mL vs. 45.6 pg/mL, LDH: 2100 IU/L vs. 948.5 IU/L, D-dimer: 4.50 mg/L vs. 3.15 mg/L, and EBV-DNA copy number: 500000 copies/ml vs. 26400 copies/ml), and the positive predictive value is also higher (Supplementary Table 1).

### Predictive models for EBV-HLH

**Fig. 4:**
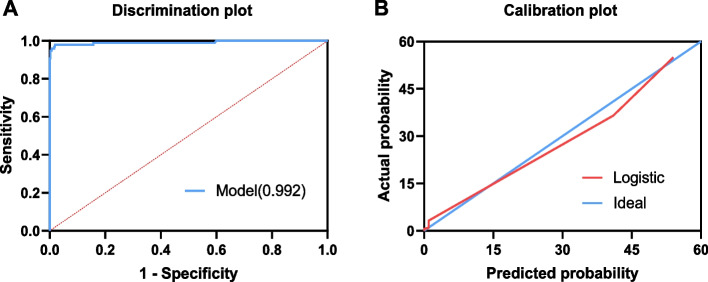
Effectiveness of the models to predict EBV-HLH A logistic regression model based on IL-10, ferritin, D-dimer and triglycerides were established. The AUC of ROC curve for this model reached 0.992 (**A**) and curves of calibration for the logistic regression model was drawn (**B**)

A single variable sometimes fails to distinguish two entities in their early stages. Thus, we developed a predictive model using multivariate logistic regression analysis to determine which factors were the most effective predictors. All the laboratory indices that were significantly different between the two groups were included, among which IL-10, ferritin, D-dimer, and triglycerides showed statistical significance in the multivariate logistic regression analysis. Then we developed a logistic regression model based on continuous variables, including IL-10, ferritin, D-dimer, and triglycerides [logit *P* = -9.969 + 0.057 × IL-10 + 0.006 × ferritin + 0.113 × D-dimer + 0.434 × triglycerides] (Table 4). The AUC for this model was 0.992 (Fig. 4A). The sensitivity and specificity were 97.96% (95% CI, 92.82–99.75) and 98.16% (96.40–99.20) for the diagnosis of EBV-HLH, and the PPV and NPV were 92.31% (85.79–95.98) and 99.53% (95% CI, 98.18–99.88), respectively. The positive and negative likelihood ratios were 53.14 (95% CI, 26.73–105.65) and 0.02 (95% CI, 0.01–0.08), respectively. Then, we employed the Hosmer–Lemeshow goodness-of-fit test to evaluate the calibration of the model, which indicated that the prediction model has a good capacity for calibration (χ^2^ = 2.077, *P* = 0.979) (Fig. 4B).

**Table 4:** Estimated model parameters in the selected multivariable logistic regression model

Parameters	Logistic regression model				
	β	SE	Wald χ^2^	*P*	OR (95% CI)
IL-10, pg/mL	0.057	0.021	7.13	0.008	1.059 (1.015,1.104)
Ferritin, g/L	0.006	0.001	25.49	< 0.0001	1.006 (1.004,1.008)
D-dimer (mg/L)	0.113	0.048	5.53	0.019	1.120 (1.019,1.123)
Triglycerides (mmol/L)	0.434	0.215	4.06	0.044	1.544 (1.012,2.355)
Constant	-9.969	2.018	24.40	< 0.0001	

Model: logit *P* = -9.969 + 0.057 × IL-10 + 0.006 × Ferritin + 0.113 × D-dimer + 0.434 × Triglyceride; The optimal cutoff probability = 0.09369, which means that if the predicted probability ≥ 0.09369, that patient is identified as high risk for EBV-HLH

Considering cytokine determination is not available or the serum cytokine levels may not return with a quick turnaround time at some medical centers for emergent situations, we established another predictive model based on physical sign and frequently used laboratory indices. Based on univariate analysis, cervical lymphadenopathy, LDH, D-Dimer and triglycerides were enrolled into a logistic regression model, The AUC for this model was 0.963[logit *P* = -6.187 + 0.002 × LDH + 0.257 × D-dimer + 0.394 × Triglyceride -3.049 × Cervical lymphadenopathy]. The sensitivity and specificity were 86.32% (95% CI, 77.98–91.83) and 96.53% (94.57–97.79) for the diagnosis of EBV-HLH (Supplementary Fig. 3 and Supplementary Table 2).

## Discussion

Most primary EBV infections present as IM in children, while a fraction of patients will progress to EBV-HLH [17]. Early management of EBV infection is related to the development of innate immune cells, such as NK (natural killer) cells and CD8 + and CD4 + T cells [17, 18]. In recent years, many studies have investigated the prognostic aspects of HLH in pediatric patients, and one of the conclusions drawn from these studies was that the lymphocyte subset was crucial for determining the disease's prognosis [19]. In addition, past research has demonstrated that the condition of children diagnosed with IM is intricately connected to the peripheral lymphocyte subsets [20]. In this study, the percentage of CD8 + T cells in EBV-HLH patients was much lower than that in EBV-IM patients, yet the levels of IFN-γ were significantly higher, indicating the complicated pathophysiology of HLH. While the percentage of CD4 + and CD8 + T cells and the ratio of CD4 + /CD8 + cells did not effectively distinguish the two entities because of their low sensitivity, similar to the findings of a previous report [21]. Immune molecular mechanisms driven by EBV infection that generate IM and HLH require further investigation.

EBV-HLH is caused primarily by EBV infection, which aberrantly activates CD8 + T and NK cells and drives their proliferation, resulting in a Th1/Th2 cell imbalance and immunological disorders with a high cytokine production [22, 23]. We previously demonstrated that the classical cytokine profile of HLH in EBV-HLH is substantially elevated IFN-γ and IL-10 and moderately elevated IL-6 [16, 24]. Here, the ROC values showed high sensitivity and specificity of the IFN-γ and IL-10 indices for recognizing EBV-HLH from EBV-IM. Therefore, it would be worthwhile to investigate the biochemical profiles of EBV-IM and EBV-HLH with different cytokine patterns following EBV infection.

Primary fibrinolysis and disseminated intravascular coagulation (DIC) can cause hypofibrinogenemia, and D-dimer is a potentially sensitive marker of intravascular fibrinolysis [25]. Patients with serious conditions such as HLH can exhibit DIC [26]. Our research revealed that patients with EBV-HLH had considerably greater D-dimer levels than those with EBV-IM, providing a valuable indicator for distinguishing the two entities. We also illustrated that the serum EBV-DNA load was significantly higher in EBV-HLH patients than in IM patients, consistent with the findings of earlier studies [6, 21, 27]; thus, if patients have a high EBV-DNA burden, the likelihood of EBV-HLH should be evaluated in depth.

In our retrospective study, some children ultimately diagnosed with HLH were misdiagnosed with IM at an earlier stage of the disease, resulting in a delay in treatment and, ultimately, death. Our results demonstrate that the PPV and NPV shift depending on the cutoff threshold used. For example, when we set the LDH cutoff value to 948.5 IU/L, the PPV is 56.2%, and the NPV is 96.6%. However, when we increased the cutoff value to 2100 IU/L, both the PPV and the NPV neared 92.0%. Thus, if a physician uses an LDH cutoff value of 948.5 IU/L as the reference, roughly 40% of children with HLH may be misdiagnosed, lowering their chances of survival. Therefore, choosing an acceptable cutoff value for clinical guidance is important.

Individuals with acute EBV infection have many clinical and biochemical characteristics of HLH, such as fever, splenomegaly, and liver dysfunction, making EBV-HLH difficult to separate from EBV-IM in some cases [28]. Although our study indicated that ferritin had good specificity and sensitivity in separating the two diseases at an early stage, some infants with HLH did not exhibit typical symptoms of elevated ferritin at that time; therefore, a comprehensive diagnosis model with multiple indicators is required. Li et al. developed an EBV-HLH score with five parameters, including hemoglobin, platelet, neutrophil, albumin, and LDH, which has a sensitivity of 89.2% and a specificity of 89.5%, to help identify EBV patients that need additional HLH screening [12]. Smits et al. discovered a minimal parameter set consisting phagocytosis, splenomegaly, cytopenia, increased ferritin, and increased triglycerides that can predict HLH with a sensitivity of 95% and a specificity of 94% [13]. However, there is no current model to distinguish EBV-HLH from IM. Based on variables (lymphadenopathy, IL-10, ferritin, D-dimer, LDH and triglycerides), we developed two predictive models for EBV-HLH in childhood with EBV infection with both specificity higher than 95%. These models may be useful to distinguish these two presentations more accurately than a single laboratory index. Interestingly, serum ferritin, which is an important parameter for the diagnosis of HLH, presented excellent ability to distinguish EBV-HLH from IM, with AUC of 0.996 (Table 3). However, considering some patients with HLH may not show significantly elevated ferritin or the result may not be quick available sometimes, other predict models still have their values in this circumstance.

Several limitations applied to this study. First, as this was a retrospective study, we could not follow up on the patients from the onset of fever to the time of EBV-HLH diagnosis. We collected the data when the diagnosis was established, thus potentially missing some biomarkers with early predictive value. Second, IL-18 is an important biomarker which is increased in infectious diseases and HLH [29]. It may have potential values for the distinguishment of HLH from IM. However, IL-18 was not included in our detection kit and we were not able to perform such analysis. Third, due to the single-center nature of this investigation, the repeatability of our results and conclusion require additional validation in other cohorts.

In conclusion, the present study showed IL-10, IFN-γ, LDH, D-dimer and ferritin were good biomarkers for distinguishing EBV-HLH from EBV-IM early. Integrating multiple biomarkers can further improve the accuracy of this model.

### Supplementary Information

**Additional file 1: Figure 1.** Laboratory indicators were compared between patients with EBV-HLH and those with EBV-IM in three age groups. (*P<0.05, **P<0.01, ***P<0.001, ****P<0.0001) A,CD4+% B,CD8+% C,CD4+/CD8+ D,IL-6 E,IL-10 F, IFN-γ G,LDH, lactate dehydrogenase H,D-dimer.

**Additional file 2: Figure 2.** The ROC curve for EBV-HLH prediction using TG, Fib, EBV-DNA loads, LDH, IL-10, IFN-γ, the D-dimer, and ferritin among three age groups. A, age ≤ 3 years old; B, > 3 years old and ≤ 6 years old; C, > 6 years old.

**Additional file 3: Figure 3.** Another logistic regression model based on cervical lymphadenopathy, LDH, D-Dimer and triglycerides were established. The AUC of ROC curve for this model reached 0.963 (A) and curves of calibration for the logistic regression model was drawn (B).

**Additional file 4: Supplementary Table 1.** Comparison of two optimal cutoff values.

**Additional file 5: Supplementary Table 2.** Estimated another model parameters in the selected multivariable logistic regression model.

## Data Availability

The datasets analyzed during the current study available from the corresponding author on reasonable request.

## Abbreviations

EBV-HLH: Epstein-Barr virus associated hemophagocytic lymphohistiocytosis
EBV-IM: Epstein-Barr virus associated infectious mononucleosis
IL-10: Interleukin-10
IFN‐γ: Interferon-gamma
LDH: Lactate dehydrogenase
Fib: Fibrinogen
