# Supplementary material for: Comparative study of biomarkers for the early identification of Epstein–Barr virus-associated hemophagocytic lymphohistiocytosis in infectious mononucleosis
Source: BMC Infect Dis. 2023 Oct 26;23:728. doi: 10.1186/s12879-023-08654-6 (PMC10601177; doi:10.1186/s12879-023-08654-6)
Supplement: Supplementary file 1 — Additional file 1: Figure 1. Laboratory indicators were compared between patients with EBV-HLH and those with EBV-IM in three age groups. (*P<0.05, **P<0.01, ***P<0.001, ****P<0.0001) A,CD4+% B,CD8+% C,CD4+/CD8+ D,IL-6 E,IL-10 F, IFN-γ G,LDH, lactate dehydrogenase H,D-dimer. [file 12879_2023_8654_MOESM1_ESM.pptx]

## Slide 1
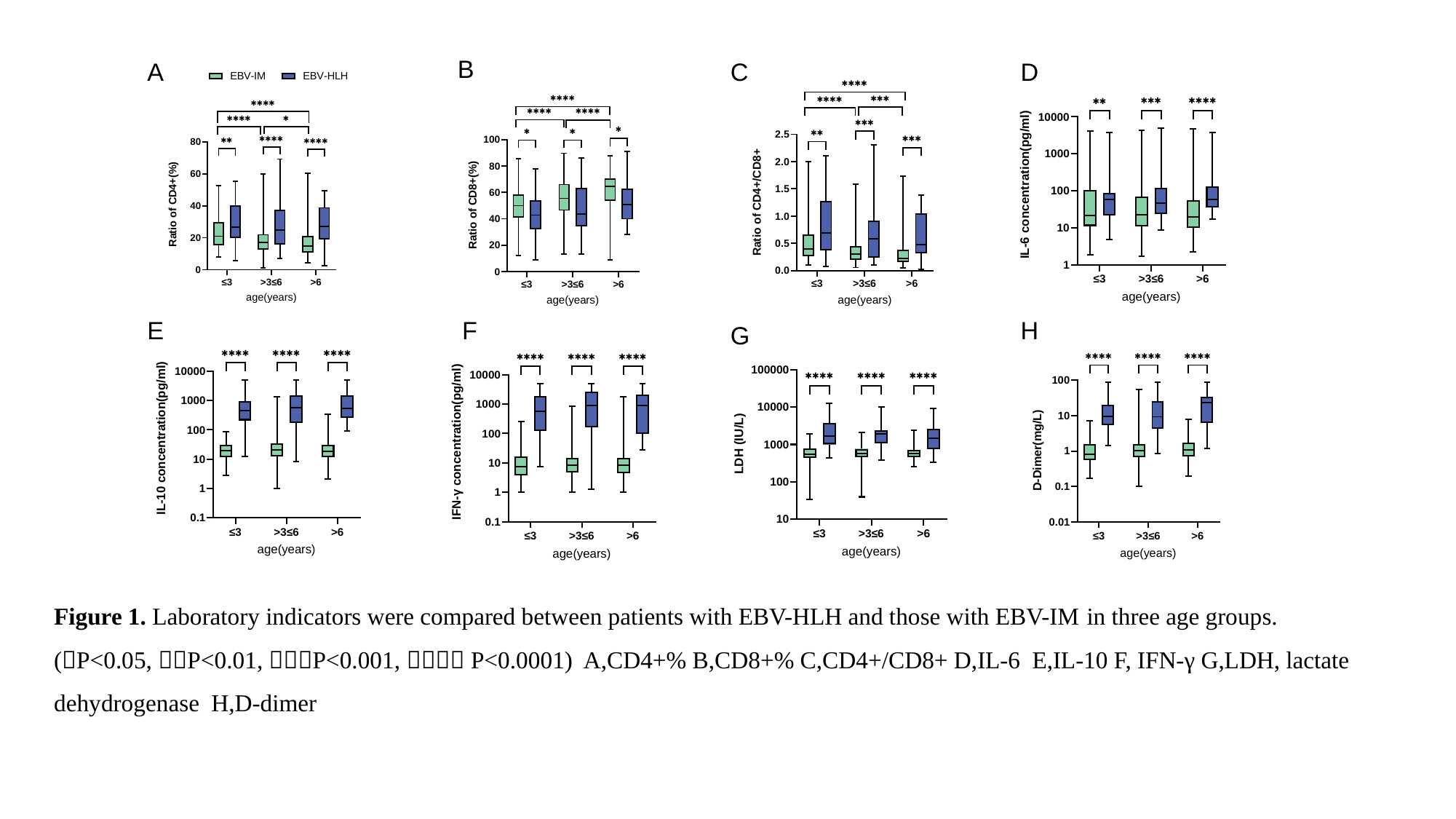

Figure 1. Laboratory indicators were compared between patients with EBV-HLH and those with EBV-IM in three age groups. (P<0.05, P<0.01, P<0.001,  P<0.0001) A,CD4+% B,CD8+% C,CD4+/CD8+ D,IL-6 E,IL-10 F, IFN-γ G,LDH, lactate dehydrogenase H,D-dimer
