# Supplementary material for: Comparative study of biomarkers for the early identification of Epstein–Barr virus-associated hemophagocytic lymphohistiocytosis in infectious mononucleosis
Source: BMC Infect Dis. 2023 Oct 26;23:728. doi: 10.1186/s12879-023-08654-6 (PMC10601177; doi:10.1186/s12879-023-08654-6)
Supplement: Supplementary file 2 — Additional file 2: Figure 2. The ROC curve for EBV-HLH prediction using TG, Fib, EBV-DNA loads, LDH, IL-10, IFN-γ, the D-dimer, and ferritin among three age groups. A, age ≤ 3 years old; B, > 3 years old and ≤ 6 years old; C, > 6 years old. [file 12879_2023_8654_MOESM2_ESM.pptx]

## Slide 1
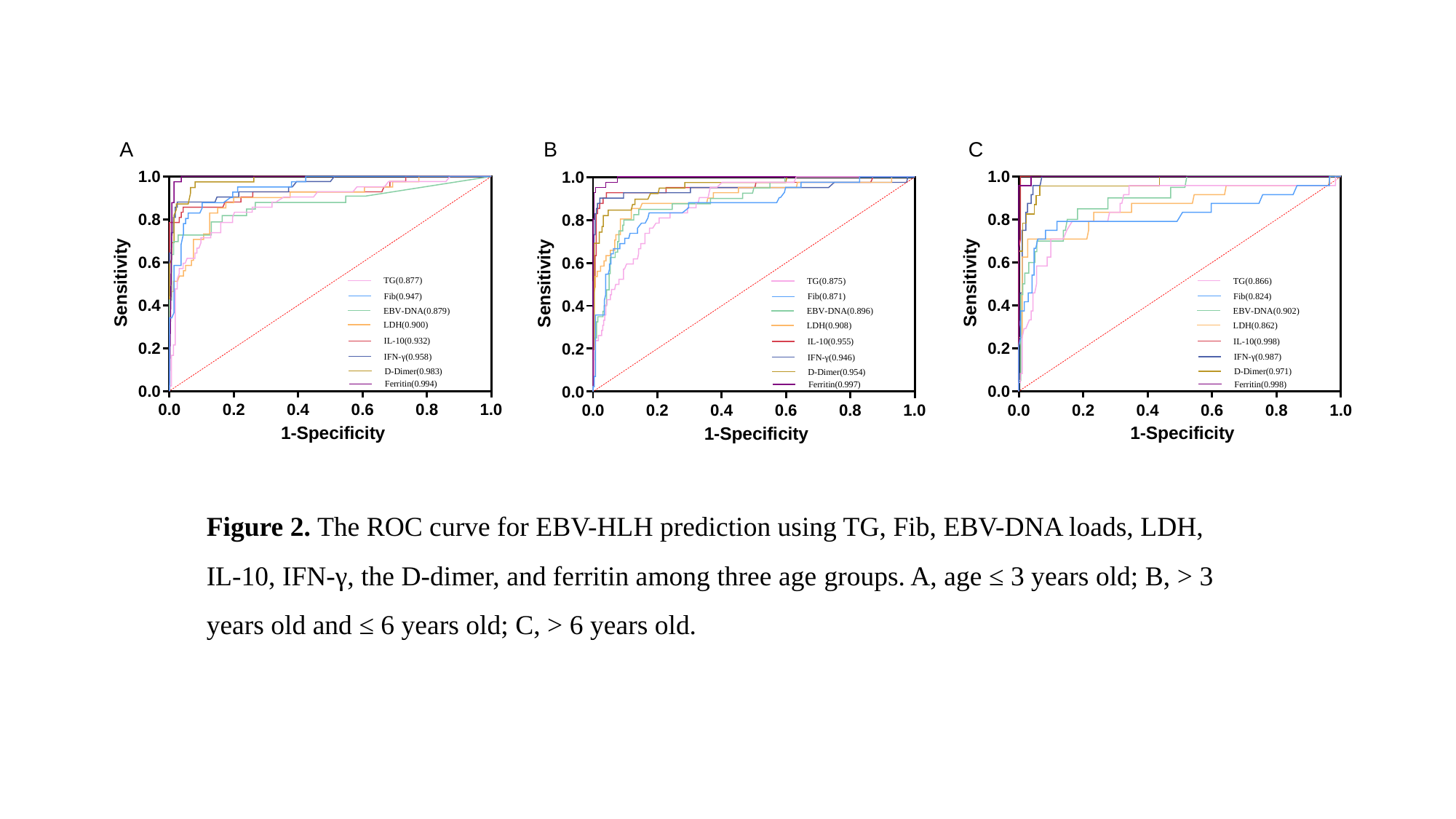

Figure 2. The ROC curve for EBV-HLH prediction using TG, Fib, EBV-DNA loads, LDH, IL-10, IFN-γ, the D-dimer, and ferritin among three age groups. A, age ≤ 3 years old; B, > 3 years old and ≤ 6 years old; C, > 6 years old.
