# Supplementary material for: Comparative study of biomarkers for the early identification of Epstein–Barr virus-associated hemophagocytic lymphohistiocytosis in infectious mononucleosis
Source: BMC Infect Dis. 2023 Oct 26;23:728. doi: 10.1186/s12879-023-08654-6 (PMC10601177; doi:10.1186/s12879-023-08654-6)
Supplement: Supplementary file 3 — Additional file 3: Figure 3. Another logistic regression model based on cervical lymphadenopathy, LDH, D-Dimer and triglycerides were established. The AUC of ROC curve for this model reached 0.963 (A) and curves of calibration for the logistic regression model was drawn (B). [file 12879_2023_8654_MOESM3_ESM.pptx]

## Slide 1
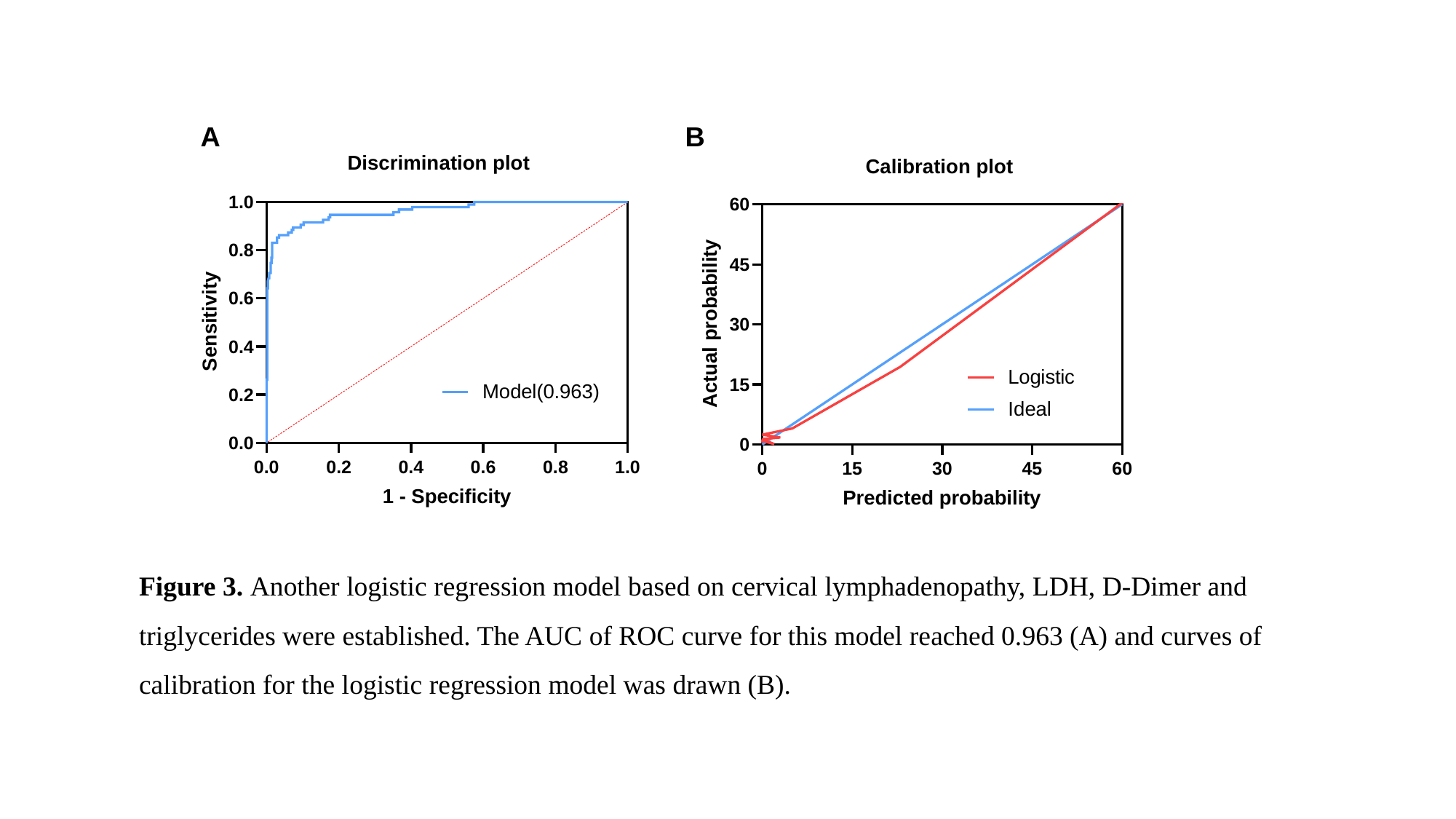

Figure 3. Another logistic regression model based on cervical lymphadenopathy, LDH, D-Dimer and triglycerides were established. The AUC of ROC curve for this model reached 0.963 (A) and curves of calibration for the logistic regression model was drawn (B).
