# Supplementary material for: Comparative study of biomarkers for the early identification of Epstein–Barr virus-associated hemophagocytic lymphohistiocytosis in infectious mononucleosis
Source: BMC Infect Dis. 2023 Oct 26;23:728. doi: 10.1186/s12879-023-08654-6 (PMC10601177; doi:10.1186/s12879-023-08654-6)
Supplement: Supplementary file 4 — Additional file 4: Supplementary Table 1. Comparison of two optimal cutoff values. [file 12879_2023_8654_MOESM4_ESM.docx]

Supplementary Table 1. Comparison of two optimal cutoff values

|  | Cutoff ^a^ | Cutoff ^b^ | PPV ^a^, % | NPV ^a^, % | PPV ^b^, % | NPV ^b^, % |
| --- | --- | --- | --- | --- | --- | --- |
| IL-10 (pg/ml) | 89.6 | 288.3 | 90.4 | 97.9 | 94.5 | 94.5 |
| IFN-γ (pg/ml) | 45.6 | 240.7 | 80.8 | 98.4 | 94.8 | 94.8 |
| LDH (IU/L) | 948.5 | 2100 | 56.2 | 96.6 | 92.0 | 92.0 |
| D-dimer (mg/L) | 3.15 | 4.50 | 73.6 | 98.3 | 90.0 | 90.0 |
| EBV-DNA load (copies/ml) | 26400 | 500000 | 59.8 | 95.8 | 89.0 | 89.0 |

^a^ the optimal Cutoff value in the maximum value Youden’s index

^b^ the optimal Cutoff value in the intersection of positive predictive value and negative predictive value
