# Supplementary material for: Comparative study of biomarkers for the early identification of Epstein–Barr virus-associated hemophagocytic lymphohistiocytosis in infectious mononucleosis
Source: BMC Infect Dis. 2023 Oct 26;23:728. doi: 10.1186/s12879-023-08654-6 (PMC10601177; doi:10.1186/s12879-023-08654-6)
Supplement: Supplementary file 5 — Additional file 5: Supplementary Table 2. Estimated another model parameters in the selected multivariable logistic regression model. [file 12879_2023_8654_MOESM5_ESM.docx]

Supplementary Table2. Estimated another model parameters in the selected multivariable logistic regression model

| Parameters | Logistic regression model | | | | | |
| --- | --- | --- | --- | --- | --- | --- |
|  | β | | SE | Wald χ^2^ | *P* | OR (95% CI) |
| LDH (IU/L) | | 0.002  0.257  0.394  -3.049  -6.187 | 0.001 | 32.168 | <0.0001 | 1.002(1.002,1.003) |
| D-dimer (mg/L) | |  | 0.064 | 15.901 | <0.0001 | 1.293(1.140,1.467) |
| Triglycerides (mmol/L) | |  | 0.127 | 9.664 | 0.002 | 1.482(1.157,1.900) |
| Cervical lymphadenopathy | |  | 0.978 | 9.713 | 0.002 | 0.047(0.007,0.322) |
| Constant | |  | 0.545 | 128.803 | <0.0001 |  |

Model: logit P = -6.187 +0.002 × LDH +0.257 × D-dimer +0.394 ×Triglyceride -3.049 × Cervical lymphadenopathy; The optimal cutoff probability = 0.1996, which means that if the predicted probability ≥ 0.1996, that patient is identified as high risk for EBV-HLH
